# Understanding O-GlcNAc transferase (OGT): Every amino acid matters

**DOI:** 10.1016/j.jbc.2025.110760

**Published:** 2025-09-24

**Authors:** Ningda Xu, Yucheng Zhao, Wei Chi, Yanqiu Yuan, Jing Li

**Affiliations:** 1 Shenzhen Eye Hospital, Shenzhen Eye Medical Center, Southern Medical University, Guangdong, China; 2 Beijing Key Laboratory of DNA Damage Response, College of Life Sciences, Capital Normal University, Beijing, China; 3 School of Pharmaceutical Sciences, Guangdong Provincial Key Laboratory of Drug Non-Clinical Evaluation and Research, Sun Yat-sen University, Guangzhou, Guangdong, China

**Keywords:** O-GlcNAc transferase, post-translational modification, O-GlcNAcase, protein–protein interaction, short linear motif

## Abstract

O-GlcNAc transferase (OGT) and O-GlcNAcase (OGA) mediate all the “writing” and “erasing” of intracellular O-GlcNAc modification events on the serine or threonine residues of proteins. Decades of investigations have revealed many O-GlcNAc substrates, spanning almost all areas of biological research. The question remains, however: why is there only one OGT? Here, we provide a tentative answer to the “one OGT” question. We propose that OGT is a sensor of various biological stimuli and responds accordingly by incurring post-translational modifications (PTMs) or through its short linear motifs (SLiMs). Both PTMs and SLiMs reside in its intrinsic disordered regions, tetratricopeptide repeats, or catalytic domains and contribute to altering its enzymatic activity, protein–protein interaction, subcellular localization, and protein stability. OGA follows the same pattern, although to a lesser extent. We propose that OGT, or OGA, can sense biological cues and, *via* its PTMs or SLiMs, adjust the downstream OGT interactome and O-GlcNAcome correspondingly.

## OGT mediates intracellular O-GlcNAcylation

**Figure 1:**
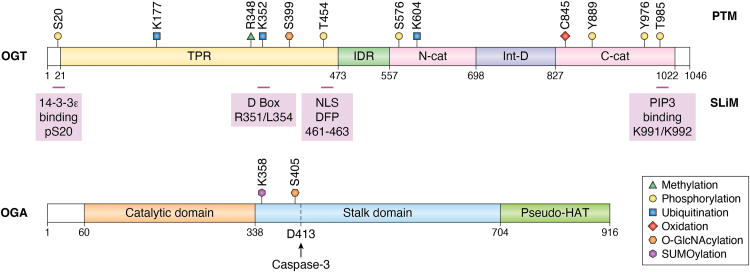
**OGT contains numerous post-translational modifications (PTMs) and short linear motifs (SLiMs) that enable it to respond to various stimuli.***Upper*, a schema showing the PTMs and SLiMs on OGT. OGT contains 13.5 tetratricopeptide repeats (TPRs) at its N terminus, followed by an intrinsically disordered region (IDR) and one catalytic (Cat) domain comprising two lobes at its C terminus. In addition, there is an intermediate domain (Int-D) between the two Cat domains (the OGT sequence is based on UniProt entry: O15294). The PTMs and SLiMs are shown. *Lower*, a schema showing the PTMs on OGA. D-box, destruction box; OGA, O-GlcNAcase; OGT, O-GlcNAc transferase; NLS, nuclear localization signal; PIP3, phosphatidylinositol 3,4,5-trisphosphate.

**Figure 2:**
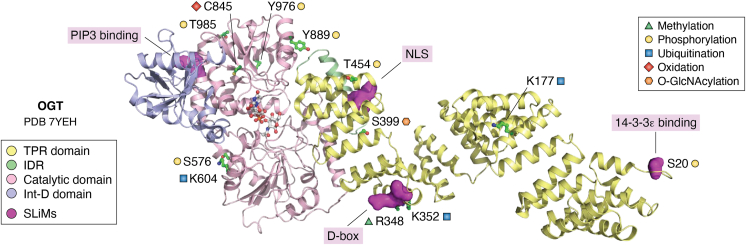
**A schematic diagram for OGT PTMs and SLiMs.** The OGT structure was extracted from the EM structure of OGT in complex with OGA (PDB code: 7YEH) using PyMOL 2.3.0. The TPR domain, IDR, catalytic domain (Cat-N and Cat-C), and the intermediate domain (Int-D) of OGT are shown in *cartoon representation* and colored in *pale yellow*, *pale green*, *light pink*, and *light blue*, respectively. The residues with reported PTMs are shown in *stick representation* with carbon atoms shown in *green* and labeled in the color scheme. The SLiMs are shown in *surface representation* and colored in *magenta*. UDP and NAG in the active site are shown in *stick representation* with carbon atoms shown in *gray*. IDR, intrinsically disordered region; OGA, O-GlcNAcase; OGT, O-GlcNAc transferase; PDB, Protein Data Bank; PTM, post-translational modification; SLiM, short linear motif; TPR, tetratricopeptide repeat.

The O-GlcNAc transferase (OGT) catalyzes all intracellular O-GlcNAcylation reactions on serine (Ser) and threonine (Thr) (1) residues. The reverse reaction is mediated by O-GlcNAcase (OGA) (1). Structural studies have revealed that it contains 13.5 tetratricopeptide repeats (TPRs) at its N terminus and one catalytic (Cat) domain comprising two lobes at its C terminus. In addition, there is an intermediate domain between the two Cat domains (Figs. 1*A* and 2). During the past 4 decades, enormous efforts have been put into identifying OGT substrates and delineating their biological functions, which conclude that about 50% of its substrates have a P-P-(V/T)-g(S)-(S/T)-A or (P/T)-P-(V/T)-g(T)-(S/T)-(A/T) motif (2). However, it does not apply to all O-GlcNAc substrates. Recently, it was reported that O-GlcNAcylation also occurs on the tyrosine (Tyr) residue based on a mass spectrometry analysis (3); however, this finding still warrants further validation.

Structurally, the 13.5 TPRs form a hydrophobic lumen where asparagine ladders (N94/N128/N165/N196/N230/N264/N298/N332/N366/N400/N434/N468) (4, 5) and aspartate ladders (D124/D162/D226/D294/D328/D396/D430/D464) (5) were identified. These asparagine and aspartate ladders line the entire TPR lumen of OGT and are required for global glycosylation, providing substrate selectivity to some degree (6). Cat activity–independent function of OGT has also been found to be associated with the TPRs, as the N-terminal 4 TPRs have been shown to bind influenza A virus genomic RNA to induce translocation of nuclear OGT to cytosolic lipid droplets, where the coating protein perilipin 2 is destabilized, thus limiting lipid droplet accumulation and viral replication (7).

By truncating the various TPRs, investigators found that the N-terminal 5 TPRs are required for OGT localization and are important for mediating many of OGT’s protein–protein interactions (6). The five TPRs are a minimum need for function in lower eukaryotes, and a human would need every TPR. This work sheds light on the substrate recognition mode of OGT, but the question remains: How can one OGT cope with all sorts of environmental insults and enable an O-GlcNAcome that is amenable to stress from both within and outside the cells? Or, why is there only one OGT?

Recent investigations are starting to provide insight into this question, as many post-translational modifications (PTMs) and a few short linear motifs (SLiMs) on OGT/OGA have been uncovered (Figs. 1 and 2) (Table 1 and 2), which may facilitate downstream changes of the O-GlcNAcome. PTMs identified on OGT so far include phosphorylation (8, 9, 10, 11, 12, 13, 14, 15, 16), O-GlcNAcylation (17), oxidation (18), methylation (19) and ubiquitination (20, 21). PTMs on OGA include O-GlcNAcylation (22) and small ubiquitin–like modifier (SUMO)ylation (23). SLiMs refer to compact interaction modules that are usually less than 10 residues in length and reside in intrinsically disordered regions (IDRs) (24). Although pivotal for binary biomolecular interactions, they are not readily amenable to systematic studies. Sometimes, it is challenging to identify functional SLiMs in a given protein. However, because of their evolutionary plasticity, they confer exceptional functional versatility and diversity. Common SLiMs include nuclear localization signals (NLSs), degrons, and kinase docking motifs. Currently, only four SLiMs have been identified on OGT, and none were identified on OGA. Both PTMs and SLiMs render OGT susceptible to outside stimuli. This article summarizes the PTMs and SLiMs on OGT and OGA. We envision that it is through PTMs and SLiMs that OGT should respond to environmental cues, which provides a molecular answer to the one OGT question.

**Table 1:** PTMs and SLiMs on OGT (UniProt entry: O15294)

	Type	Sites	Function	Conserved?^a^	References
PTMs	Phosphorylation	S20	Binding with 14-3-3ε, stabilization	Conserved	(8, 9, 10, 11)
		T454	Chromatin disassociation	Conserved	(12, 13)
		S576	Stabilization	Conserved	(14)
		Y889, T985	Activity	Y889 not conserved in fly, T985 conserved	(15)
		Y976	Substrate selectivity	Conserved	(16)
	O-GlcNAcylation	S399	Nuclear localization	Conserved	(17)
	Oxidation	C845	Increasing OGT activity toward FOXK2	Conserved	(18)
	Methylation	R348	Stabilization	Conserved	(19)
	Ubiquitination	K177	Degradation	Conserved	(20)
		K352	Binding with Cdc27	Conserved	(21)
SLiMs	14-3-3 binding	pS20	Stabilization	Conserved	(11)
	D-box	R351/L354	Binding with Cdc20	Not conserved in fly or worm	(21)
	NLS	DFP (aa 461–463)	Nuclear localization	Conserved	(17)
	PIP3 binding	K991/K992	PI(3,4,5)P3 binding, plasma membrane localization	Not conserved in fly or worm	(37)

a The sequences were compared among human, mouse, rat, worm, and fly.

**Table 2:** PTMs on OGA (UniProt entry: O60502)

	Type	Sites	Function	Conserved?^a^	References
PTMs	O-GlcNAcylation	S405	Inhibiting ubiquitin-mediated degradation	Not conserved in fly or worm	(22) (45) (46)
	SUMOylation	K358	Chaperone-mediated autophagy	Conserved	(23)
	Caspase cleavage	D413	Cleaved into two fragments	Not conserved in fly	(49)

a The sequences were compared among human, mouse, rat, worm and fly.

## PTMs and SLiMs on OGT

### PTMs on OGT

#### Phosphorylation governs OGT stability, localization, and substrate selectivity

##### OGT is phosphorylated at S20 by checkpoint kinase 1 and calmodulin-dependent protein kinase II for stabilization

Lying before the TPRs is the N-terminal tail IDR of OGT that comprises 20 amino acids. In 2011, a phosphoproteome screen of checkpoint kinase 1 (CHK1) inadvertently identified OGT as one of its substrates, with S20 being phosphorylated (25). Further work validated that S20 is indeed phosphorylated by CHK1, which promotes OGT abundance and its localization to the midbody (8). It was demonstrated that phospho-S20 (pS20) occurs precisely during the later stages of mitosis and mediates the intermediate filament bridge severing (8). Recent work further uncovered that pS20 creates a SLiM for 14-3-3ε binding (11), which is one member of the phospho-binding signal adaptor/scaffold 14-3-3 protein family. As the 14-3-3 family is known to promote protein stability (26), 14-3-3ε binding may contribute to stabilizing OGT by pS20.

Interestingly, pS20 is also shown to be a pivotal target by other kinases, that is, calcium–calmodulin-dependent protein kinase II (CaMKII) (9). Upon glucagon stimulation, liver autophagy is induced as a metabolic adaptation to nutrient depletion. During this process, glucagon induces calcium signaling to activate CaMKII, which phosphorylates OGT at S20, thereby stabilizing OGT and increasing O-GlcNAcylation levels to activate Unc-51-like autophagy activating kinase 1 (ULK1) for autophagy initiation. This mechanism is highly related to diabetes and related human disorders. Both CHK1 and CaMKII share a consensus motif of R-X-X-S/T, and other common substrates have also been identified for these two kinases, for example, STN1 (27). Later investigations demonstrate that pS20 is also conserved in *Drosophila* and mouse embryonic stem cells and embryonic fibroblasts, where it regulates DNA damage response and maintains gut homeostasis, implicating OGT in stem cell–derived diseases (10).

##### OGT is phosphorylated at T454 by AMP-activated protein kinase for nuclear export

In 2014, two independent studies reported that AMP-activated protein kinase (AMPK) phosphorylates OGT. One article found that AMPK phosphorylates OGT at T454 for chromatin disassociation (12), thereby inhibiting downstream histone H2BS112 O-GlcNAcylation and gene transcription. In another study, AMPK also phosphorylates OGT at T454, which inhibits OGT nuclear localization and histone H3K9 acetylation in C2C12 human skeletal muscle myotubes, affecting downstream O-GlcNAcome (13). T454-phosphorylated OGT has recently been reported to bind robustly with the transcription factor NRF2 and promote lung cancer malignancy (28). Therefore, the interactome and O-GlcNAcome vary as OGT is modified in different patterns.

##### Glucose deprivation promotes OGT phosphorylation at S576 by ULK1 for stabilization

Previously, three independent studies reported that OGT levels increase after prolonged glucose depletion or fasting, and the potential mechanism was proposed to be an increase in OGT mRNA (29, 30, 31). A recent report (14) found that glucose deprivation increases the interaction between OGT and ULK1, which is a kinase that is activated when glucose is scarce. Using *in vitro* kinase assay and mass spectrometry, OGT is found to be phosphorylated in a ULK1-dependent manner at S576, which stabilizes OGT by increasing the affinity between OGT and its deubiquitinase BRCA1-associated protein 1 (BAP1) (14). It was further found that OGT-S576A markedly decreased tumorigenesis in colon cancer xenograft models. Thus, a ULK1–OGT–BAP1 axis is in play when glucose is deprived.

##### High glucose induces OGT phosphorylation at Y889 and T985 in glioblastoma

PI3Kβ usually functions as a lipid kinase. In glioblastoma cells upon high glucose conditions, OGT is phosphorylated by hexokinase 1 at Y889, which recruits the p85α subunit of PI3K (15). Subsequently, PI3Kβ moonlights as a protein kinase to phosphorylate OGT at T985 to enhance OGT enzymatic activity (15). In turn, ATP-citrate synthase (ACLY) is O-GlcNAcylated, and CoA production is elevated, resulting in more robust fatty acid levels and histone H3 acetylation for gene transcription in glioblastoma (15).

##### OGT phosphorylation at Y976 is promoted by epidermal growth factor for substrate selectivity

The C terminus of OGT is also subject to phosphorylation. Epidermal growth factor promotes OGT Y976 phosphorylation, which enhances the affinity between OGT and phospho-tyrosine binding proteins, such as pyruvate kinase M2 (PKM2) (16). Consequently, PKM2 O-GlcNAcylation is upregulated, resulting in detetramerization and a decrease in PKM2 activity (16). Notably, Y384 and Y844 are also found to be phosphorylated in an epidermal growth factor–dependent manner, albeit to a lesser degree (16). Thus, pY976 provides another paradigm where phosphorylation creates a binding motif (*i.e.*, phosphotyrosine) for other proteins and leads to a changing O-GlcNAcome.

#### Glucose promotes OGT methylation by coactivator-associated arginine methyltransferase 1 at R345 for stabilization

In non–small cell lung cancer (NSCLC) cells, glucose promotes the arginine methylation of OGT (19). By screening the potential arginine methyltransferases (CARM1, PRMT5, PRMT6, and PRMT7), the authors identified coactivator-associated arginine methyltransferase 1 (CARM1) as the catalyzing enzyme. Further mass spectrometry and mutagenesis studies pinpointed R345 as the modified residue. Biochemical assays revealed that R345 methylation promotes OGT stability by its deubiquitinase USP9X. By elevating cellular O-GlcNAcylation, OGT R345 methylation upregulates c-Myc expression and promotes NSCLC tumorigenesis (19). It is interesting that in this study, glucose deprivation leads to lower OGT levels, and glucose abundance induces higher OGT levels in NSCLC cells, whereas OGT-pS576 is found to stabilize OGT upon glucose deprivation (14). Perhaps glucose concentration affects OGT levels in a cell type–specific manner.

#### Oxidation at C845 activates OGT activity toward Forkhead Box K2 upon oxidative stress

Previously, three independent investigations reported that O-GlcNAcylation levels would increase upon ferroptosis or oxidative stress (32, 33, 34). A quantitative proteomics study examined the OGT interactome upon hydrogen peroxide stress and found that many proteins change their association with OGT (34). For instance, host cell factor 1 dissociates from OGT in a time-dependent manner after oxidative stress (34).

A recent report revealed that treating hepatocellular carcinoma cells with a ferroptosis inducer (*e.g.*, erastin or RSL3) increases OGT activity, while not affecting OGT abundance or UDP-GlcNAc levels (18). Mass spectrometry identified that OGT is oxidized (sulfinylated) at C845 and activated. Then OGT O-GlcNAcylates Forkhead Box K2 (FOXK2), a transcription factor, and promotes FOXK2 nuclear import and binding to the promoter of solute carrier family 7 member 11 (SLC7A11). As SLC7A11 is indispensable for the uptake of extracellular cystine by most cancer cells, an increase in SLC7A11 transcription upregulates cystine uptake and reduces lipid reactive oxygen species production (18). Thus, an OGT–FOXK2–SLC7A11 axis protects against ferroptosis. It would be interesting to explore whether C845 oxidation would also enhance OGT activity to other substrates during ferroptosis.

#### OGT ubiquitination modulates its abundance

##### OGT is ubiquitinated at K177, which is reversed by ubiquitin-specific peptidase 8 in hepatocellular carcinoma

The deubiquitinase ubiquitin-specific peptidase 8 (USP8) is found to inhibit cancer cell proliferation and induce cell ferroptosis in hepatocellular carcinoma (20). Interestingly, USP8 is phosphorylated by SLK at S716 and reversed by the protein phosphatase PPP1CA. S716 phosphorylation increases the binding between USP8 and OGT. Through bioinformatics and mutagenesis studies, OGT Lys177 is found to be the ubiquitination site targeted by USP8 (20). Stabilized OGT subsequently O-GlcNAcylates SLC7A11 for cystine import into the cell. This study provides another example where OGT stabilization alters its downstream substrates. As OGT is known to interact with PPP1C, we wonder whether other PPP1C-interacting proteins, especially deubiquitylating enzymes and ubiquitin E3 ligases, would have the same effect on OGT abundance.

##### OGT is ubiquitinated at K352 to enhance OGT binding with anaphase-promoting complex/cyclosome^Cdc20^

OGT has long been found to be downregulated during mitosis (35). Recently, OGT has been shown to be ubiquitinated by the mitotic E3 ligase anaphase-promoting complex/cyclosome (APC/C)^Cdc20^ (21). Specifically, OGT K352 ubiquitination will prime OGT binding with CDC27, a subunit of APC/C. K352 lies right in the middle of the destruction box (D-box), a SLiM recognized by Cdc20, thus entailing OGT degradation by APC/C^Cdc20^ (21). This finding provides another example that PTMs crosstalk with SLiMs. It also sheds light on the underlying mechanism of why protein O-GlcNAcylation antagonizes CDK1-mediated phosphorylation, as OGT stability is in decline when CDK1-mediated phosphorylation is on the rise during mitosis.

### Auto O-GlcNAcylation of OGT mediates its nuclear localization

Besides phosphorylation, OGT is also subject to auto-O-GlcNAcylation, as its plant homolog, SECRET AGENT (SEC), also auto O-GlcNAcylates itself (36). OGT S399 is found to be O-GlcNAcylated, where it lies juxtaposing to its noncanonical NLS DFP (amino acids 461–463) (17). By associating with importin α5, OGT O-GlcNAcylation promotes its nuclear import through NLS. The potential NLS is among the first SLiMs reported on OGT. The crosstalk between O-GlcNAcylation and NLS exemplifies a typical theme of regulation: the synergy between PTMs and SLiMs.

The NLS finding also warrants further discussion, as DFP lies in an RTALKLKPDFPDAY stretch on OGT, where the upstream positively charged residues may be part of the NLS. There is also a predicted NLS C terminal to DFP. Therefore, the DFP mutation may disrupt the NLS. Moreover, it is worth noting that this stretch is located in the TPR region, rendering its interaction with importin α5 rather challenging. Future investigations on TPRs and PTMs on OGT may help resolve this dilemma. Further questions remain: are there any other NLSs on OGT? If so, are they also mediated by importin α5?

### OGT contains four SLiMs

SLiMs are abundant in IDRs and are fundamental for signal transduction (24). Often, they crosstalk with PTMs, offering versatility in modulating protein functions. Although SLiMs are quite abundant in cells, relatively few SLiMs have been reported on OGT. In fact, currently there are only four: pS20, which creates a SLiM for 14-3-3ε binding (please refer to the previous phosphorylation section); the potential NLS (please refer to the previous O-GlcNAcylation section); a D-box degron; and a lipid-interacting SLiM. In addition, OGT interacts with a SLiM through its own IDR.

#### OGT contains a D-box amenable to cell cycle control

The D-box refers to the conserved “R x x L” motif. Proteins harboring the D-box are susceptible to interacting with the ubiquitin E3 ligase APC/C and being controlled by the cell cycle. By mutating and screening all the potential D-boxes in OGT, R351–L354 was found to be the degron that is essential for binding to Cdc20, a coactivator of APC/C (21). Mutating the D-box will result in mitotic defects and inhibit uterine carcinoma, suggestive of the physiological role of the SLiM in OGT.

#### A SLiM mediates OGT–lipid interaction and OGT localization

SLiMs mediate not only protein–protein interaction but also OGT–lipid interaction (37). The basic amino acids K991/K992 of OGT are found to interact with phosphatidylinositol 3,4,5-trisphosphate, which is pivotal for insulin signaling. Consequently, OGT is recruited to the plasma membrane upon serum stimulation, where it enhances insulin receptor substrate (IRS1)-S307 and IRS1-S632/635 phosphorylation, while attenuating AKT-T308 phosphorylation, thus perturbing glucose and lipid metabolism (37).

#### OGT binds SLiMs

Not only does OGT contain SLiMs but also OGT itself binds to SLiMs (38, 39). Recent work using phage display identified that the intermediate domain region of OGT binds to regulators with SLiMs. One work found the (Y/F)-x-P-x-Y-x-(I/M/F) motif, and the other work found P-x-Y-x-(I/L) (38, 39). Importantly, phosphorylation of the Tyr in the SLiM attenuates affinity with OGT, implicating Tyr phosphorylation in regulating OGT. It is unknown whether other regions of OGT also bind SLiMs.

#### Outlooks on SLiMs

With only four SLiMs reported on OGT so far, a coherent view on them is yet to form. However, it is likely that they also affect OGT stability and localization. In fact, the 14-3-3ε-interacting SLiM augments OGT stability, whereas the D-box mediates OGT mitotic degradation. The lipid-interacting motif directs the plasma membrane localization of OGT, and the NLS modulates nuclear localization of OGT. More SLiMs identified in the future may reveal the underlying mechanisms.

It might be a common theme that SLiMs crosstalk with PTMs, as they often occur in the IDR. Indeed, three of the four SLiMs neighbor PTMs (Figs. 1 and 2): pS20 creates a SLiM for 14-3-3ε binding; O-GlcNAcylation promotes its interaction with importin α5 *via* its NLS; ubiquitination at K352 enhances its binding with Cdc20 (one coactivator of APC/C) *via* the D-box. Perhaps, it is worthwhile to explore the vicinity of PTMs to look for potential SLiMs.

AlphaFold is instrumental for IDR prediction. It has been incorporated with bioinformatic tools to score putative SLiM plausibility in *Leishmania* (40). In other studies, AlphaFold has been efficient for *de novo* modeling of SLiMs that interact with other known protein domains. For instance, AlphaFold redefined the proline C degron on the DNA polymerase subunit POLD2 on FEM1B (41), demonstrating great potential for *de novo* SLiM prediction *in silico*. The combination of AlphaFold and experimental results will surely identify more SLiMs on OGT.

## PTMs on OGA

OGA consists of an N-terminal hydrolase domain, a stalk domain in the middle, and a C-terminal pseudo histone acetyltransferase (HAT) domain (Fig. 1*B*) (1). Investigations have demonstrated that the pseudo-HAT domain is essential for OGA recruitment to DNA damage sites upon laser microirradiation (42). A full-length structure of OGA was recently revealed by cryo-EM, and histone array experiments found that the pseudo-HAT domain binds to methylated H3K36 peptides and H4 acetylated peptides, indicating a role of OGA as a reader of the histone code (43). As these histone marks are associated with DNA damage repair, perhaps these two observations are interlinked.

Compared with OGT, fewer PTMs were identified on OGA. Below, we will delineate the O-GlcNAcylation and SUMOylation on OGA. We believe that with the advent of cutting-edge mass spectrometry instruments, more PTMs will be identified on OGA.

### OGA is O-GlcNAcylated at S405 for degradation

As early as 2006, OGA was shown to associate with OGT in a stable transcriptional complex (44). Later, OGA was found to be O-GlcNAcylated at S405 by electron-transfer dissociation mass spectrometry (22). A recent cryo-EM structural study also confirmed that OGT and OGA form an autoinhibitory complex and that a GlcNAc moiety is covalently attached to OGT S405 (45). As far as its function is concerned, S405 O-GlcNAcylation modulates OGA stability (46), which is commonly observed in O-GlcNAcylated substrates. It is possible that OGA O-GlcNAcylation alters its affinity with ubiquitin E3 ligases, as OGA has been demonstrated to be ubiquitinated and degraded by E3 ligases, such as TRIM33 (47) and the nuclear ligase Ubiquitin protein ligase E3 component n-Recognin 5 (UBR5) (48). It is also worth investigating whether S405 O-GlcNAcylation entails other functional changes on OGA.

### OGA is cleaved by caspase-3 at D413 during apoptosis

During apoptosis, OGA is cleaved at D413 by caspase-3 at a non-canonical SVVD recognition site (49). The cleavage does not affect the glycosidase activity of OGA, as both fragments associate with each other, and they can reconstitute hydrolase activity in cells (49). Considering that O-GlcNAcylation plays an important role in apoptosis (50), does the cleaved OGA fragment display activity changes toward specific apoptotic proteins? Since D413 is in close proximity to S405, could they crosstalk so that the cleavage affects OGA O-GlcNAcylation?

### OGA is SUMOylated at K358 for chaperone-mediated autophagy

A previous proteomic screen identified that OGA is subject to the SUMO modification (www.phosphosite.org) (51). Further mutagenesis studies revealed that OGA is SUMOylated at K358 (23), promoting its binding with the SUMO-interaction motif (SIM) of heat shock cognate protein 70, a chaperone protein for the chaperone-mediated autophagy (CMA) pathway. Subsequently, OGA is shunted to the CMA pathway for degradation.

Intriguingly, a label-free quantitative mass spectrometry analysis using the heat shock cognate protein 70-SIM mutant revealed many potential CMA client proteins that might also be SUMOylated (23), suggesting that the SUMO–SIM binding between CMA client proteins and chaperones may be a common theme in the CMA pathway. The authors validated the model by studying the SUMOylation of a histone reader, YEATS2, and its subsequent degradation by CMA (23). Whether such a lock-and-key mechanism holds for other CMA substrates warrants further investigation.

## Concluding remarks

In conclusion, OGT is amenable to various environmental cues. Although there is only one OGT, PTMs and SLiMs provide a handle for stress signals to act on OGT, allowing OGT to alter its localization, abundance, enzymatic activity, and substrate selectivity. Consequently, the downstream OGT interactome and O-GlcNAcome may change accordingly. As the PTMs and SLiMs do not rely on the transferase activity of OGT, they are not typically found in glycomics studies, which often leave their discoveries by chance. By studying the PTMs and SLiMs on OGT, we may uncover new biology on OGT, thus answering the “one OGT” question.

Open questions remain in the field. Currently, O-GlcNAcome studies using these OGT PTM and SLiM mutants are lacking, partly because of technical constraints. Quantitative proteomics has been used to examine the OGT interactome upon oxidative stress and has revealed substantial differences in OGT-interacting proteins after hydrogen peroxide treatment (34). Perhaps the same rationale can be used to unearth the O-GlcNAcome variations in the future. Since it has long been appreciated that O-GlcNAc adjusts to various nutrient statuses, then what kinds of PTMs would occur on OGT and OGA in response to disparate nutrient stimuli? Does OGA harbor SLiMs as well? Does OGA also bind to SLiMs? The advent of various techniques, including click chemistry, mass spectrometry, and AlphaFold, sheds a beckoning light. By delineating PTMs and SLiMs on OGT and OGA, the jigsaw puzzle of OGT will unfold.

## Conflict of interest

The authors declare that they have no conflicts of interest with the contents of this article.

## References

[1] Cheng S.S., Mody A.C., Woo C.M. (2024). Opportunities for therapeutic modulation of O-GlcNAc. Chem. Rev..

[2] Wulff-Fuentes E., Berendt R.R., Massman L., Danner L., Malard F., Vora J. (2021). The human O-GlcNAcome database and meta-analysis. Sci. Data.

[3] Hou C., Deng J., Wu C., Zhang J., Byers S., Moremen K.W. (2024). Ultradeep O-GlcNAc proteomics reveals widespread O-GlcNAcylation on tyrosine residues of proteins. Proc. Natl. Acad. Sci. U. S. A..

[4] Levine Z.G., Fan C., Melicher M.S., Orman M., Benjamin T., Walker S. (2018). O-GlcNAc transferase recognizes protein substrates using an asparagine ladder in the Tetratricopeptide Repeat (TPR) superhelix. J. Am. Chem. Soc..

[5] Joiner C.M., Hammel F.A., Janetzko J., Walker S. (2021). Protein substrates engage the lumen of O-GlcNAc transferase's tetratricopeptide repeat domain in different ways. Biochemistry.

[6] Potter S.C., Gibbs B.E., Hammel F.A., Joiner C.M., Paulo J.A., Janetzko J. (2024). Dissecting OGT's TPR domain to identify determinants of cellular function. Proc. Natl. Acad. Sci. U. S. A..

[7] Dong H., Liang C., Zhang J., Wu W., Kumar N., Liu Z. (2025). O-GlcNAc transferase plays dual antiviral roles by integrating innate immunity and lipid metabolism. Nat. Commun..

[8] Li Z., Li X., Nai S., Geng Q., Liao J., Xu X. (2017). Checkpoint kinase 1-induced phosphorylation of O-linked beta-N-acetylglucosamine transferase regulates the intermediate filament network during cytokinesis. J. Biol. Chem..

[9] Ruan H.B., Ma Y., Torres S., Zhang B., Feriod C., Heck R.M. (2017). Calcium-dependent O-GlcNAc signaling drives liver autophagy in adaptation to starvation. Genes Dev..

[10] Na H.J., Akan I., Abramowitz L.K., Hanover J.A. (2020). Nutrient-driven O-GlcNAcylation controls DNA damage repair signaling and stem/progenitor cell homeostasis. Cell Rep..

[11] Yan S., Yuan K., Yao X., Chen Q., Li J., Sun J. (2024). 14-3-3epsilon augments OGT stability by binding with S20-phosphorylated OGT. J. Biol. Chem..

[12] Xu Q., Yang C., Du Y., Chen Y., Liu H., Deng M. (2014). AMPK regulates histone H2B O-GlcNAcylation. Nucleic Acids Res..

[13] Bullen J.W., Balsbaugh J.L., Chanda D., Shabanowitz J., Hunt D.F., Neumann D. (2014). Cross-talk between two essential nutrient-sensitive enzymes: o-glcnac transferase (OGT) and AMP-activated protein kinase (AMPK). J. Biol. Chem..

[14] Lv Z., Da Q., Li Y., Yuan A., Shao G., Lu X. (2025). ULK1-dependent phosphorylation of OGT instructs the tumorigenicity of O-GlcNAcylation. Sci. China Life Sci..

[15] He X., Chen D., Liu G., Wu Q., Zhao H., Guo D. (2025). PI3Kbeta functions as a protein kinase to promote cellular protein O-GlcNAcylation and acetyl-CoA production for tumor growth. Mol. Cell.

[16] Wang Y., Shu H., Liu J., Jin X., Wang L., Qu Y. (2022). EGF promotes PKM2 O-GlcNAcylation by stimulating O-GlcNAc transferase phosphorylation at Y976 and their subsequent association. J. Biol. Chem..

[17] Seo H.G., Kim H.B., Kang M.J., Ryum J.H., Yi E.C., Cho J.W. (2016). Identification of the nuclear localisation signal of O-GlcNAc transferase and its nuclear import regulation. Sci. Rep..

[18] Zhang H., Ma J., Hou C., Luo X., Zhu S., Peng Y. (2025). A ROS-mediated oxidation-O-GlcNAcylation cascade governs ferroptosis. Nat. Cell Biol..

[19] Lin L., Yuan Q., Gu J., Bai G., Cong X., Hu Q. (2024). CARM1-mediated OGT arginine methylation promotes non-small cell lung cancer glycolysis by stabilizing OGT. Cell Death Dis..

[20] Tang J., Long G., Hu K., Xiao D., Liu S., Xiao L. (2023). Targeting USP8 inhibits O-GlcNAcylation of SLC7A11 to promote ferroptosis of hepatocellular carcinoma via stabilization of OGT. Adv. Sci. (Weinh).

[21] Meng L., Dong R., Mi W., Qin K., Ouyang K., Sun J. (2024). The ubiquitin E3 ligase APC/C(Cdc20) mediates mitotic degradation of OGT. J. Biol. Chem..

[22] Khidekel N., Ficarro S.B., Clark P.M., Bryan M.C., Swaney D.L., Rexach J.E. (2007). Probing the dynamics of O-GlcNAc glycosylation in the brain using quantitative proteomics. Nat. Chem. Biol..

[23] Yan S., Yuan A., Shao G., Zhou W., Xu X., Dong M.Q. (2025). SUMOylation targets O-GlcNAcase to chaperone-mediated autophagy. J. Biol. Chem..

[24] Kumar M., Michael S., Alvarado-Valverde J., Zeke A., Lazar T., Glavina J. (2024). ELM-the Eukaryotic linear motif resource-2024 update. Nucleic Acids Res..

[25] Blasius M., Forment J.V., Thakkar N., Wagner S.A., Choudhary C., Jackson S.P. (2011). A phospho-proteomic screen identifies substrates of the checkpoint kinase Chk1. Genome Biol..

[26] Pennington K.L., Chan T.Y., Torres M.P., Andersen J.L. (2018). The dynamic and stress-adaptive signaling hub of 14-3-3: emerging mechanisms of regulation and context-dependent protein-protein interactions. Oncogene.

[27] Jaiswal R.K., Lei K.H., Chastain M., Wang Y., Shiva O., Li S. (2023). CaMKK2 and CHK1 phosphorylate human STN1 in response to replication stress to protect stalled forks from aberrant resection. Nat. Commun..

[28] Zhang Y., Sun C., Ma L., Xiao G., Gu Y., Yu W. (2024). O-GlcNAcylation promotes malignancy and cisplatin resistance of lung cancer by stabilising NRF2. Clin. Transl. Med..

[29] Kang J.G., Park S.Y., Ji S., Jang I., Park S., Kim H.S. (2009). O-GlcNAc protein modification in cancer cells increases in response to glucose deprivation through glycogen degradation. J. Biol. Chem..

[30] Taylor R.P., Parker G.J., Hazel M.W., Soesanto Y., Fuller W., Yazzie M.J. (2008). Glucose deprivation stimulates O-GlcNAc modification of proteins through up-regulation of O-linked N-acetylglucosaminyltransferase. J. Biol. Chem..

[31] Cheung W.D., Hart G.W. (2008). AMP-activated protein kinase and p38 MAPK activate O-GlcNAcylation of neuronal proteins during glucose deprivation. J. Biol. Chem..

[32] Zhang C.C., Li Y., Jiang C.Y., Le Q.M., Liu X., Ma L. (2024). O-GlcNAcylation mediates H(2)O(2)-induced apoptosis through regulation of STAT3 and FOXO1. Acta Pharmacol. Sin..

[33] Yu F., Zhang Q., Liu H., Liu J., Yang S., Luo X. (2022). Dynamic O-GlcNAcylation coordinates ferritinophagy and mitophagy to activate ferroptosis. Cell Discov..

[34] Martinez M., Renuse S., Kreimer S., O'Meally R., Natov P., Madugundu A.K. (2021). Quantitative proteomics reveals that the OGT interactome is remodeled in response to oxidative stress. Mol. Cell Proteomics.

[35] Saunders H., Dias W.B., Slawson C. (2023). Growing and dividing: how O-GlcNAcylation leads the way. J. Biol. Chem..

[36] Shrestha R., Karunadasa S., Grismer T.S., Reyes A.V., Xu S.L. (2024). SECRET AGENT O-GlcNAcylates hundreds of proteins involved in diverse cellular processes in arabidopsis. Mol. Cell Proteomics.

[37] Yang X., Ongusaha P.P., Miles P.D., Havstad J.C., Zhang F., So W.V. (2008). Phosphoinositide signalling links O-GlcNAc transferase to insulin resistance. Nature.

[38] Alteen M.G., Meek R.W., Kolappan S., Busmann J.A., Cao J., O'Gara Z. (2023). Phage display uncovers a sequence motif that drives polypeptide binding to a conserved regulatory exosite of O-GlcNAc transferase. Proc. Natl. Acad. Sci. U. S. A..

[39] Blankenship C.M., Xie J., Benz C., Wang A., Ivarsson Y., Jiang J. (2023). Motif-dependent binding on the intervening domain regulates O-GlcNAc transferase. Nat. Chem. Biol..

[40] Tusnady G.E., Zeke A., Kalman Z.E., Fatoux M., Ricard-Blum S., Gibson T.J. (2023). LeishMANIAdb: a comparative resource for leishmania proteins. Database (Oxford).

[41] Timms R.T., Mena E.L., Leng Y., Li M.Z., Tchasovnikarova I.A., Koren I. (2023). Defining E3 ligase-substrate relationships through multiplex CRISPR screening. Nat. Cell Biol..

[42] Cui Y., Xie R., Zhang X., Liu Y., Hu Y., Li Y. (2021). OGA is associated with deglycosylation of NONO and the KU complex during DNA damage repair. Cell Death Dis..

[43] Nyenhuis S.B., Steenackers A., Hinshaw J.E., Hanover J.A. (2025). Human O-GlcNAcase catalytic-stalk dimer anchors flexible histone binding domains. Res. Square.

[44] Whisenhunt T.R., Yang X., Bowe D.B., Paterson A.J., Van Tine B.A., Kudlow J.E. (2006). Disrupting the enzyme complex regulating O-GlcNAcylation blocks signaling and development. Glycobiology.

[45] Lu P., Liu Y., He M., Cao T., Yang M., Qi S. (2023). Cryo-EM structure of human O-GlcNAcylation enzyme pair OGT-OGA complex. Nat. Commun..

[46] Gorelik A., Bartual S.G., Borodkin V.S., Varghese J., Ferenbach A.T., van Aalten D.M.F. (2019). Genetic recoding to dissect the roles of site-specific protein O-GlcNAcylation. Nat. Struct. Mol. Biol..

[47] Kweon T.H., Jung H., Ko J.Y., Kang J., Kim W., Kim Y. (2024). O-GlcNAcylation of RBM14 contributes to elevated cellular O-GlcNAc through regulation of OGA protein stability. Cell Rep..

[48] Du Y., Yang Z., Shi H., Chen Z., Chen R., Zhou F. (2024). E3 ubiquitin ligase UBR5 promotes gemcitabine resistance in pancreatic cancer by inducing O-GlcNAcylation-mediated EMT *via* destabilization of OGA. Cell Death Dis..

[49] Butkinaree C., Cheung W.D., Park S., Park K., Barber M., Hart G.W. (2008). Characterization of beta-N-acetylglucosaminidase cleavage by caspase-3 during apoptosis. J. Biol. Chem..

[50] Seyrek K., Ivanisenko N.V., Konig C., Lavrik I.N. (2024). Modulation of extrinsic apoptotic pathway by intracellular glycosylation. Trends Cell Biol..

[51] Lumpkin R.J., Gu H., Zhu Y., Leonard M., Ahmad A.S., Clauser K.R. (2017). Site-specific identification and quantitation of endogenous SUMO modifications under native conditions. Nat. Commun..

